# Assessment of factors affecting practice towards COVID-19 among health care workers in health care facility of West Guji Zone, South Ethiopia, 2020

**DOI:** 10.11604/pamj.2021.39.53.27798

**Published:** 2021-05-19

**Authors:** Shiferaw Gelchu Adola, Girish Degavi, Sarah Ezhil Kelna Edwin, Takala Utura, Udessa Gemede, Pandiarajan Kasimayan

**Affiliations:** 1 Bule Hora University´s Department of Nursing, College of Health and Medical Science, Hagere Mariam, Ethiopia,; 2 Bule Hora University´s Department of Midwifery, College of Health and Medical Science, Hagere Mariam, Ethiopia,; 3 Bule Hora University´s Department of Public Health, College of Health and Medical Science, Hagere Mariam, Ethiopia

**Keywords:** Prevention, COVID-19, health care workers, West Guji Zone

## Abstract

**Introduction:**

health care workers are at greatest risk to being infected with COVID-19 in health care facilities. This study is focused on assessing the level of practice and factors affecting practice towards COVID-19 among health care workers in health care facility of West Guji Zone, Oromia region, Ethiopia.

**Methods:**

health facility based cross-sectional study design was carried out from December 1^st^ to 30^th^ 2020 among health care providers in West Guji Zone. The simple random sampling technique was used in study and total sample size for this study was 281. The data enter into Epi Data version 4.4.3.1 and SPSS Version 25 used for analysis. The descriptive statistics and logistic regression are needed. The cut point for statically significance settled at p < 0.05.

**Results:**

the response rate in this study was 97.8%. Of all study participants, 50.2%, 8.4%, and 6.5% had traveling history, chronic illnesses, and contact with COVID-19 confirmed cases. Too much working, lack of training, and shortage of protective equipment were reported by 54.5%, 50.9%, and 29.1% respectively. About 36.4% and 38.2% of health care providers had poor level of knowledge and prevention practice towards COVID-19. Working at hospital (AOR= 0.156, 95% CI=0.033-0.741), awareness of the action during suggestive symptoms and signs of COVID-19 developed (AOR= 0.038, 95% CI=0.002-0.817), hand washing (AOR= 0.043, 95% CI=0.008-0.238), not going to crowded place (AOR= 0.001, 95% CI=0.001-0.030), applying physical distance (AOR=0.091,95% CI=0.041-0.579) adherence to triage and isolation protocol (AOR=0.317,95%=0.039-0.577)and knowledge level of COVID-19 (AOR=2.378,95%CI=1.523-6.322) were factors significantly associated with prevention practice of COVID-19.

**Conclusion:**

in this study, the knowledge level and prevention practice gap was identified. Type of facility, awareness of the action during suggestive symptoms and signs of COVID-19 developed, hands washing to the standard, not going to crowded place, keeping physical distance, adherence to triage and isolation protocol and having good level of knowledge about COVID-19 were factors associated with good prevention practices. Adequate supply of personal protective materials; provision of continuous on-job training and guideline for prevention of COVID-19 must be given to all health care facilities.

## Introduction

The severe acute respiratory syndrome corona virus-2 (SARS-CoV-2) causes mild to severe respiratory disease called COVID-19 disease that is mainly transmitted through respiratory droplets and contact of materials with causative agents [1-3]. The corona virus outbreak occurred several times in the world. However, the current outbreak due to SARS-CoV-2 virus results several deaths across the globe and currently a public health issues for developed and developing countries [3, 4]. Health care workers are risk groups for COVID-19 disease in the health care facility than the general population because of their responsibility in response to the SARS-CoV-2 virus epidemic and they are front line people to contact patients [4-6]. According to Amnesty International and World Health Organization reports, the number of health care providers infected with SARS-CoV-2 virus and died due to COVID-19 disease is higher in European continent [7-9]. In African countries, the health care delivery system was below the standard even before the happening of COVID-19 outbreak. The deficiencies in the system can be explained in terms of inadequate health work forces, material resources, underfunded, and healthcare management information systems [8,10]. Even though it is the challenge, health workers should access with personal protective equipment such as masks, gloves, gowns, and other personal protective equipment (PPE) required to do their jobs safely and effectively. Lack of education in dealing with COVID-19 cases, high number of patients flow, longer period of working, and insufficient rest periods were some of the factors attributed to infections at health care facility [5,6,11] The protective materials needed to protect health care workers from COVID-19 outbreak vary based on the type of tasks to be performed, working environments, and risk to contact with suspected or confirmed cases. Health care providers who are in contact with confirmed cases, particularly physicians, nurses, allied health, and supportive staffs who providing services at all levels of health care delivery system should strictly apply standard precaution packages and be able to understand that personal protective equipment is essential strategies in prevention and control of COVID-19 disease [12-17]. It is important to identify the level of knowledge and practice regarding COVID-19 among health care workers. The aims of this study is to assess prevention practice towards COVID-19 and associated factors among health care workers in health facilities of West Guji Zone which enables to set future plan for intervention and provide information for all stakeholders who are able to give solution to the problem.

## Methods

**Study area**: the study was conducted in the selected health care facilities of West Guji Zone, which is located in Southern Ethiopia, Oromia regional state. The zone was found at the southern direction from the capital city Addis Ababa. Based on the 2007 census conducted by the Central Statistical Agency of Ethiopia (CSA), there are 1,300,000 populations living in the West Guji Zone that is 50.4% males and 49.6% females. There are 42 health facilities found in West Guji Zone, in which three of them are hospitals that provide health care services to community of the zone and surrounding areas.

**Study design**: health facility-based cross-sectional study design was conducted among health care workers working in the selected health care facilities of West Guji Zone. Study population: the study populations were health care workers currently working in the selected health care facilities in West Guji Zone. Health care workers currently working in the selected health care facilities in West Guji Zone that were available during the data collection period included in this study. Health care workers who were sick and unable to fill the questionnaires were excluded from this study.

**Sample size determination**: sample size was determined based on objectives. The first objective was to determine sample size using standard formula for single population proportion based on the following assumptions. Z (α/2) =1.96 (95% confidence level of the survey), 62% prevalence from the previous study, and 5% margin of error was used in this study to give sample size 362. The study population was less than 10,000 and correction formula needed. The sample size after adding 10% of non-respondents was 281 for objective one. For second objective sample size calculated by EPi Info using three independent variables and the EPi Info output after adding 10% of non-respondents were less than the sample from objective one. Therefore, the final sample size for this study was that of the objective one, which is n=281. Simple random sampling technique was used to include study participants in the study.

**Measurements**: to measure the knowledge of health care workers, 16 questions were used. The correct answer was assigned 1 point and an incorrect answer was assigned 0 points. The total practice score ranged from 0 to 16. Health care workers who correctly answered 75% or more of the practice items were considered as having good knowledge and those scores less than < 75 categorized as poor knowledge towards COVID-19 [11]. To measure the prevention practices of health care workers, 11 questions were used. The correct answer was assigned 1 point and an incorrect answer was assigned 0 points. The total practice score ranged from 0 to 11. Health care workers who correctly answered 75% or more of the practice items were considered as having good prevention practice and those scores less than < 75 categorized as poor prevention practice towards COVID-19 [11].

### Operational definition

**Health care workers**: HCWs are defined as all people engaged in activities whose primary intention is to improve health. In this study, health care workers includes; Medical Doctors, Nurses, Midwifes, Health officers, Lab Technicians, and Pharmacists.

**Good practice**: in this study, health care workers who scored points greater than or equal to75% out of 11 prevention practice assessing questions.

**Poor practice**: health care workers who scored points less than 75% out of 11 practice assessing questions.

**The data collection tools and techniques**: the data collection tools adapted by reviewing different relevant articles. The data were collected using self-administered questionnaire technique. The questionnaire divided into five parts: part I: questions about socio-demographic factors, part II: personal and source of information assessing questions, part III: health facility related factors assessing questions, part IV: knowledge related questions and part V: practice assessing questions. It was prepared in English language and was reviewed by the language experts. Trained data collectors involved data collection and the data collection procedures were closely supervised.

**Quality control**: the quality of the data secured by standard, pre-tested questionnaires, proper data collection procedure, and reliability test. Before the actual data collection, pre-testing was done on 10% of the total study subjects in the Yabalo Hospital which was not included in the actual study and based on the findings, necessary amendments were made. The prevention practice assessing questions were tested statistically for internal consistency (reliability) using Cronbach´s alpha test. The overall Cronbach´s alpha value for the questionnaire to assess prevention practice towards COVID-19 was 0.754 which indicates that the items were somewhat inter-related.

**Data processing and analysis**: the data were checked for completeness; data were entered into Epi Data version 4.4.3.1 and exported to SPSS Version 25 for analysis. The results of descriptive statistics were summarized and presented by tables, charts, and graphs. Bivariate logistic regression analysis was done to check the association between dependent and independent variables. Then variables that had an association at p< 0.25 were entered in to multivariate logistic regression analysis. Statically significance was settled at p < 0.05 and 95% confidence intervals (CI).

**Ethical clearance:** it was taken from Bule Hara University, College of Health and Medical Science, Institutional Review Board (IRB). Permission letter was given to health care managers and written consent´ taken from each participant by explaining the objective of the study before the data collection and voluntarily participation of the study participates ensured in this study. The finding of the study was disseminated to all concerned bodies via presentation and publication. Therefore, in order for the participants to benefit from the study, the copy of the final report of the study was given to the two hospitals and eight health centers of the West Guji Zone. Additionally, the study will be published on peer reviewed scientific journals and disseminated to different nationally and international stake holders.

## Results

**Socio-demographic variables of health care workers in West Guji Zone, 2020**: self-administered questionnaire were distributed to 281 healthcare workers. However, 275 of them responded the questionnaires. The response rate in this study was 97.8%. More than half of the study participants were males 61.8% and the mean age of the study subjects was 27.7 (+ 3.7). Majority of health care workers were nurses 38.45% and more than two-thirds of them were living in urban. Fifty-five point six percent (55.6%) of participants were married in terms of marital status and 61.1% were degree holders followed by diploma. Concerning their family size, majority 65.45% of them live with family members. Greater than two-thirds of health care workers 76.4% had work experience less than or equal to five years and 56.4 % works in the hospital (Table 1).

**Table 1:** socio-demographic variables of health care workers study done in the West Guji Zone, 2020

Variables	Category	Frequency(N)	Percent (%)
**Sex**	Female	170	61.8
	Male	105	38.2
**Age**	<25	100	36.36
	25-35	167	60.7
	>35	8	2.9
**Profession**	Physician	25	9.05
	Nurse	103	37.45
	Health officer	27	9.8
	Midwifery	45	16.4
	Laboratory	38	13.8
	Pharmacist	37	13.5
**Residence**	Rural	68	24.7
	Urban	207	75.3
**Marital Status**	Single	122	44.4
	Married	153	55.6
**Education level**	Diploma	80	29.1
	Degree	168	61.1
	Master degree and above	27	9.8
**Type of health facility**	Health center	120	43.6
	Hospital	155	56.4
**Number of family members**	≤ 4	180	65.45
	>4	95	34.54
**Service Year**	≤ 5 years	210	76.4
	>5 years	65	23.6

**Personal and source of information of health care workers in West Guji Zone, 2020**: from all study participants, half 50.2% of them have travelled within the past two months. Ninety-one point six percent (91.6%) of HCWs were free from chronic illnesses. However, 8.4% of those who had chronic illnesses were at risk of poor outcome of COVID-19 disease. Only 6.5% of HCWs had contact with COVID-19 confirmed cases. All most all of the health care personnel were nonsmokers. However, 18.2% of them were alcohol drinkers. Regarding internet access, the majority of health professionals were used of Wifi and internet data. Eighty-one point eight percent (81.8%) of HCWs were used of TV or radio for further access to information about COVID-19 and nearly half of them use social media sometimes, in addition to TV and radio for update themselves (Table 2).

**Table 2:** personal and source of information of health care workers in the West Guji Zone, 2020

Variables	Category	Frequency(N)	Percent (%)
**Do you have travel history within two months**	Yes	138	50.2
	No	137	49.8
**Do you have chronic illnesses (DM, kidney disease, heart diseases, hypertension?**	Yes	23	8.4
	No	252	91.6
**Do you have close contact with confirmed cases**	Yes	18	6.5
	No	257	93.5
**Do you smoke cigarette in any amount**	Yes	3	1.1
	No	272	98.9
**Do you take alcohol in any amount**	Yes	50	18.2
	No	225	81.8
**Do you have internet access (wifi or mobile data)**	Yes	228	82.9
	No	47	17.1
**Do you have TV and/ or radio**	Yes	225	81.8
	No	50	18.2
**Do you use consultation of seniors for COIVID-19 virus diagnosis and further update**	Always	35	12.7
	Occasionally	182	66.2
	Never	58	21.1
**Do you use social media for information access about COVID-19**	Always	127	46.1
	Occasionally	133	48.4
	Never	15	5.5

**Health facility related factors among health care workers in West Guji Zone, 2020**: of all respondents, more than half of HCWs reported there were adequate facemasks, sanitizers, disinfectants, and have a guideline in their working area. However, the number of participants that reported in adequacy of COVID-19 prevention materials in the health facility was not minimal. Greater (29.1%) proportion of health professionals report the shortage of personal protective equipment, which is the main factor that lowers the prevention practice of COVID-19. The mean working hours per week among HCWs in West Guji Zone was 47 (+ 9). Training on infection prevention as well as COVID-19 prevention was the crucial step. Half (50.9%) of HCWs in West Guji Zone did not got training on COVID-19 prevention in this year (Table 3).

**Table 3:** health facility related factors among health care workers in the West Guji Zone, 2020

Variables	Category	Frequency(N)	Percent (%)
Do you have adequate facemask in your facility	Yes	177	64.4
	No	98	35.6
Do you have adequate hand rub sanitizer in your facility	Yes	175	63.6
	No	100	36.4
Do you have adequate disinfectant in your facility	Yes	170	61.8
	No	105	38.2
Do you have infection prevention guideline/manual in your working area	Yes	168	61.1
	No	107	38.9
Which of the following lower your prevention practice of COVID-19 virus infection?	Shortage of protective equipment	80	29.1
	High work load for long periods	42	15.3
	Negligence from my friends/staff	55	20
	Poor quality of protective equipment	40	14.5
	Discomfort while wearing PPE	40	14.5
	More than two answers	18	6.6
Total working hours/week	≤40	125	45.5
	>40	150	54.5
Did you get training on infection prevention practices in this year	Yes	135	49.1
	No	140	50.9

**COVID-19 knowledge of health care workers at West Guji Zone, 2020**: majority (96.4%) of health care workers were correctly differentiated the causative agent of COVID-19 disease and (38.9%) participants responded source of COVID -19 disease correctly. Ninety-five point three percent (95.3%) and 96.4% of HCWs were reported as respiratory droplets and contaminated objects were the main routes of COVID-19 transmission. More than half of the health professionals (65.5%) told that elderlies and patients with underlying chronic disease were the most risk groups of COVID-19 and (84.4%) of them agree that asymptomatic carriers can transmit the disease. All most all of HCWs agree on frequent hand washing 90.9%, maintaining social distance 97%, timely isolation of potentially risky cases 97%, and wearing facemask 93.5% were the important methods of tackling the spread of COVID-19 (Table 4). The 175 (63.6%) of health care providers had good level of knowledge about COVID-19 whereas 100 (36.4%) had poor knowledge level.

**Table 4:** knowledge of health care workers about Covid-19virus s in the West Guji Zone, 2020

Variables	Category	Frequency(N)	Percent (%)
**Respiratory droplets during coughing, sneezing from an infected person and close contact with infected person are main transmission routes of COVID-19**	True	262	95.3
	False	5	1.8
	I do not know	8	2.9
**Contaminated objects and surfaces can transmit COVID-19**	True	265	96.4
	False	3	1.1
	I do not know	7	2.5
**Do you think asymptomatic carriers in subclinical stage can spread the disease?**	Yes	232	84.4
	No	13	4.7
	I don’t know	30	10.9
**There is currently no effective treatment or vaccine for COVID-19, but early symptomatic and supportive treatment can help most patients recover from the infection**	True	245	89.1
	False	8	2.9
	I do not know	22	8
**Frequent hand washing with water, soap and alcohol-based hand rub sanitizer prevent COVID-19 infection**.	True	250	90.9
	False	25	9.1
**Keeping social distance as per the standard prevent VOVID-19 virus infection**.	True	267	97
	False	8	3
**Timely isolation of potentially risky/or confirmed people is important to prevention COVID-19 virus infection**	True	267	97
	False	8	3
**Wearing facemask or shields is important to prevent acquiring COVID-19 virus infection**	True	257	93.5
	False	18	6.5
**Do you know how to use and dispose personal protective equipment?**	Yes	253	92
	No	22	8
**Polymerase chain reaction (PCR) is a diagnostic tool to COVID-19 virus infection**	True	198	72
	False	25	9.1
	I don’t know	52	18.9
**I know the measures what should do if you develop symptoms and signs suggestive of COVID-19?**	Yes	248	90.2
	No	27	9.8

**Prevention practice towards COVID-19 among HCWs at West Guji Zone, 2020**: in this study, 170 (61.8) of the health care workers had good prevention practice and 105 (38.2%) had poor prevention practice towards COVID-19. Nearly one-third of HCWs were inappropriately disposes used tissue paper and had never gone to crowded places. Greater than two-thirds of respondents were practicing social distance (77.5%), routinely disinfecting the working room (88.4%), and strictly applying triage and isolation of suspected COVID-19 cases 83.6% (Table 5).

**Table 5:** practice of health care workers towards COVID-19 virus in the West Guji Zone, 2020

Variables	Category	Frequency(N)	Percent (%)
**Do you cover your mouth and nose with elbow or tissue or handkerchief?**	Yes	248	90.2
	No	27	9.8
**Do you throw the tissue you use safely in a dustbin?**	Yes	202	73.5
	No	73	26.5
**Do you use frequent hand washing with water and soap/or alcohol-based bund rub sterilizer as per recommended?**	Always	180	65.5
	Occasionally	95	34.5
**Do you routinely wear a facemask or shields at work and outside working places**	Always	153	55.6
	Occasionally	117	42.6
	Never	5	1.8
**Do you wear gloves when you were engaged in patient management**	Always	153	55.6
	Occasionally	117	42.6
	Never	5	1.8
**Do not go to the crowded places**	Always	73	26.6
	Occasionally	172	62.5
	Never	30	10.9
**Do not practice hand shaking or shoulder kissing**	Always	112	40.7
	Occasionally	118	42.9
	Never	45	16.4
**Do you avoid touching your eyes, nose or mouth as far as you can?**	Always	130	47.3
	Occasionally	127	46.2
	Never	18	6.5
**Are you practicing social distancing recommended by the WHO and CDC (2 meters)?**	Yes	213	77.5
	No	62	22.5
**I routinely disinfect of tables, surfaces and working room before and after managing patients**	True	243	88.4
	False	32	11.6
**I have strictly followed a protocol for triage and isolation of suspected COVID-19 cases in my workplace?**	True	230	83.6
	False	45	16.4

**Factors associated with prevention practice towards Covid-19 among health care workers in West Guji Zone, 2020**: factors associated with prevention practice toward COVID-19 disease were assessed using logistic regression model based on the conceptual framework. Both binary and multivariable logistic regression was done. Variables which reached P-value of less than 0.25 were considered as a candidate to multivariable logistic regression. Statistical significance was adjusted for multivariable logistic regression at P-value less than 0.05. Accordingly, the independent variables that had an association with the prevention practice of COVID-19 was: type of health facility, health care workers who were working at the hospital were 84.4% more likely to be protected from COVID-19 than those who works at health center. Study participants who had awareness about sign and symptoms of COVID-19 were 96% more likely protected from COVID-19. Study subjects who frequently wash their hands as per the recommended standard were 95.7% more likely exercised good practice to wards COVID-19. Additionally, limiting going to a crowded place, avoiding hand shaking, maintaining social distancing, and strictly following the triage protocol and isolation of suspected COVID-19 cases were factors significantly associated with good practice of COVID-19. Health care workers who had a good level of knowledge about COVID-19 were two times more likely applying the prevention practice COR: 2.378 95%, CI (1.523-6.322) than the counterpart (Table 6).

**Table 6:** factors associated with prevention practice towards COVID-19 among health care workers in the West Guji Zone, 2020

Variables	Level practice		COR With 95% CI	AOR With 95% CI	P values
	**Good**	**Poor**			
Type of health facility					
Health center	90	30	1	1	
Hospital	80	75	0.356(0.156-0.809)	0.156(0.033-0.741)	0.017*
Polymerase chain reaction (PCR) is a diagnostic tool to COVID-19 virus infection					
Yes	135	62	0.285(0.105-0.775)	1.051(0.035-1.437)	0.116
No	35	43	1	1	
I know the measures what should do if you develop symptoms and signs suggestive of COVID-19?					
Yes	165	82	0.111(0.023-0.544)	0.038(0.002-0.817)	0.037*
No	5	23	1	1	
Do you throw the tissue you use safely in a dustbin?					
Yes	138	65	0.384(0.161-0.915)	0.281(0.054-1.457)	0.131
No	32	40	1	1	
Do you use frequent hand washing with water and soap/or alcohol-based bund rub sterilizer as per recommended?					
Always	143	38	0.107(0.043-0.264)	0.043(0.008-0.238)	0.000*
Semi times	27	67	1	1	
Do not go to the crowded places					
Always	67	5	0.007(0.001-0.082)	0.001(0.001-0.026)	0.000*
Occasional	100	72	0.066(0.008-0.539)	0.009(0.012-0.149)	0.001*
Never	3	28	1	1	
Do not practice hand shaking or shoulder kissing					
Always	110	3	0.001(0.00001-0.047)	0.001(0.001-0.030)	0.000*
Occasional	52	65	0.045(0.063-0.971)	0.207(0.038-1.116)	0.067
Never	3	37	1	1	
Are you practicing social distancing recommended by the WHO and CDC (2 meters)?					
Yes	155	58	0.117(0.042-0.330)	0.091(0.041-0.579)	0.031*
No	15	47	1	1	
I have strictly followed a protocol for triage and isolation of suspected COVID-19 cases in my workplace?					
Yes	153	77	0.323(0.114-0.916)	0.317(0.039-2.577)	0.282*
No	17	28	1	1	
Knowledge level of health care workers					
Good	105	70	4.08(3.602-10.810)	2.378(1.523-6.322)	0.011*
Poor	65	35	1	1	

Key: COR- crude odds ratio, AOR- adjusted odds ratio, * -Significant at P value <0.05

## Discussion

This study focused on the factors affecting the prevention practice of SARS- CoV-2 virus among health care workers in West Guji Zone. The health care providers were at greater risk of COVID-19 disease as compared to the general population because they were involved in direct client care at health care facilities (weather hospitals or health centers) [9,14,15].The SARS- CoV-2 viruses need greater concern of infection prevention strategies to reduce its transmission and to overcome the infection of COVID-19 at the health care facilities [4,16]. The magnitude of health care workers having good prevention practice towards COVID-19 were 170 (61.8) in this study. The result is lower than studies done in Iran 71.3% and Greece 73.8% [18,19]. This magnitude is in line with cross-sectional study conducted in and in northern Ethiopia, Amara regional state (62%), Cameroon (60.8%), and Ethiopia (63.5%) [11,20,21]. However, this finding is also higher than cross-sectional study carried out at southern Ethiopia, Gamo zone 35.3% [22]. The observed difference might be due to the study setting, study population, study time, and awareness level of study participants regarding the implementation of COVID-19 prevention protocol. As this study, the percentages of HCWs who used PPE were 153 (55.6%). The result is comparable 56.8% with the similar study done at South Ethiopia gamo zone [22]. This may be happen due to the similarity in socio-demographics characteristics and geographical location. This finding was lower than studies conducted in China 98%, Cameroon 100%, and Ethiopia 67.3% [19,21,23]. The difference might be due to the study period, the method needed in data collection, types of tools used for data collection, and study setting difference. Alcohol based hand rub (ABHR)/ sanitizer, 70% alcohol, biohazard bag, 0.5% Chlorine Solution that required avoiding the infection of COVID-19 in the healthcare facility [16]. In this study, the implementation of correct hand washing as per recommended was 180 (65.5%). The result was in line with study carried out in southern Ethiopia, gamo zone 68.9% [22]. This finding lower than studies done in Cameroon (94.5%), among residents of Ethiopia 84% [24], in northern Ethiopia Amara regional state 96.1% [11], and in Libya 97.8% [25]. The difference can be explained in terms of population size variation, time of study carried out, and methodology followed.

Maintaining physical distance is an important way of talking about spread of SARS- CoV-2 virus in the health care facilities. The numbers of health care workers keeping physical distance in this were 213 (77.5%). This is similar to the study conducted in Cameroon 83.8% [20]. However, higher than the survey carried out among residents of Ethiopia 61% [24]. This variation may be occurred due to study population, sample size, and study period that the sample size of the previous study was greater and this was done during the epidemic controlled. Health care workers must apply all appropriate measures to control the transmission of SARS- CoV-2 virus; needs safe work place and limiting going to crowed place [12]. According to this survey, the proportion of health care providers avoiding going to crowd places was 73 (26.6%). This finding is not in agreement with studies conducted in northern Ethiopia, Amara regional state 54%, Cameroon 96.4%, Ethiopia 40.2%, and Tigre region 49.8% [11,20,24,26]. The main reason for in agreement may be the difference in study population, study area coverage, and study time which the previous study done during an epidemic raised. During the time epidemic was raised, information related to COVID-19 virus was disseminated through social media as well as mass media and different stake holders participated in awareness creation concerning COVID-19 virus prevention. The result of the present study shows that, the magnitude of health care workers who have chronic illness, the use of substance like smoking, and drinking alcohols were 8.4%, 1.1%, and 18.2% respectively. This finding is lower than finding from previous study done in Amara region, northern Ethiopia which the percentage of health care providers having chronic illness, smoking , and drinking alcohol was 15%, 12%, and 45% respectively [11]. The difference may be due to study area, sample size, method of the data collection and study period. Thus, the previous study area involves a wider coverage area and the sample size was larger than this study. Previous study used on line data collection technique and it was done during lock-down period.

According to the present study, the magnitude of health care providers that had good level of knowledge about COVID-19 was 63.6%. This result is a comparable with cross-sectional study done in North West Ethiopia 64.1% and Southern India 63.49% [27,28]. The finding was lower than studies conducted in northern Ethiopia, Amara region 70%, Ethiopia 88.2%, Libya 89.3%, Egypt 80.4%, South East Nigeria 88.59%, Nigeria during outbreak 88.75%, North central Nigeria 99.5%, Nepal 76%, and a systematic review in Iran 72.2% [11,21,25,29-34]. This finding is higher than studies carried out in Greece 55% and South Africa 24% [19,35]. The variation may be due to study setting, study population size, the study period, and the knowledge score cut point difference. Factors associated with good practice of COVID-19 prevention were assessed by binary and multivariable logistic regression models. Accordingly, the independent variables that had an association with the prevention practice of COVID-19 were: type of working facility, awareness of the measures on what should be done if they develop symptoms and signs suggestive of COVID-19 virus, frequently washing hand as per recommended standard, limiting going to crowded place, avoiding hand shaking, maintaining social distancing and strictly following triage protocol and isolation of suspected COVID-19 cases. Health care workers at the hospital were 84.4% times more likely protected from COVID-19 than those work at in the health center. This result is supported by a study done in northern Ethiopia, Amara region that HCWs from health centers were 60% times less likely to have good COVID-19 prevention practice [11]. This is due to the fact that working in hospital allows access to update information about COVID-19 as compared to the health center. In the present study, health care providers who have a good level of knowledge about COVID-19 were two times more likely applying prevention practice COR: 2.378 95%, CI (1.523-6.322) than HCWs that had poor level of knowledge. This result is in agreement with the study conducted in North Ethiopia, Amara regional state, Greece, and Nepal [11,19,33]. This can be explained due to the fact that having adequate knowledge of COVID-19 supports prevention practices. The limitation of this study is from the design, cross-sectional study design was used in this survey that catches data at a single point of a given time and may not be accurate as time progresses. Observational check lists were appropriate ways of assessing practice. However, in this study, practice was assessed by self-administrated questionnaires.

## Conclusion

In this study, the knowledge level and prevention practice gap were identified in study area which indicates needs of training; in way that health care workers protect themselves and the others from SARS- CoV-2 virus. Shortage of personal protective equipment was the main factor affecting prevention practice from SARS- CoV-2 virus. Thus, all health care facilities ensure continuous and adequate supply of personal protective materials. Type of facility, awareness of the action during suggestive symptoms and signs of COVID-19 developed, hand washing to the standard, not going to crowded place, keeping physical distance, adherence to triage and isolation protocol, and having good level of knowledge about COVID-19 were factors associated with good prevention practice. Therefore, all health care workers should implement COVID-19 prevention protocol.

### What is known about this topic

- SARS- CoV-2 virus is causative agent of COVID-19 disease, which was becoming a global public health issues and result several health, psychosocial, and economic problems;
- Health care workers were front line in COVID-19 outbreaks response and they were risk group to the disease;
- The COVID-19 disease was highly contagious and health care setting was favorable place for its transmission.

### What this study adds

- This study identifies the level of knowledge and practice gap among health care workers in the study area;
- Shortage of COVID-19 protective equipment and improper implementation of COVID-19 protocol was identified in this study;
- This study put base line for future plan of intervention and gives information to all concerned bodies that are able to provide solution to the problem.
